# Not what it looks like: mate-searching behaviour, mate preferences and clutch production in wandering and territory-holding female fiddler crabs

**DOI:** 10.1098/rsos.160339

**Published:** 2016-08-24

**Authors:** M. Peso, E. Curran, P. R. Y. Backwell

**Affiliations:** Research School of Biology, The Australian National University, Canberra, Australian Capital Territory 0200, Australia

**Keywords:** mate-searching, fiddler crab, wandering females, deception, sensory bias

## Abstract

Risks inherent in mate-searching have led to the assumption that females moving sequentially through populations of courting males are sexually receptive, but this may not be true. We examined two types of fiddler crab females: wanderers moving through the population of courting males and residents that were occupying and defending their own territories. Sometimes residents leave territories to look for new burrows and we simulated this by displacing wanderers and residents and observing their behaviour while wandering. We predicted that the displaced wanderers would exhibit more mate-searching behaviours than resident females. However, wandering and resident females behaved nearly identically, displaying mate-searching behaviours and demonstrating matching mate preferences. Also, males behaved the same way towards both female types and similar proportions of wanderers and residents stayed in a male's burrow to mate. But more wanderers than residents produced egg clutches when choosing a burrow containing a male, suggesting females should be categorized as receptive and non-receptive. Visiting and rejecting several males is not the defining feature of female mate choice. Moving across the mudflat by approaching and leaving a succession of burrows (mostly occupied by males) is an adaptive anti-predator behaviour that is useful in the contexts of mate-searching and territory-searching.

## 1. Introduction

Extended female mate-searching can facilitate the choice of a good quality male, but the behaviour is risky. Searching females can be exposed to predators, heat stress, dehydration and other dangers. The high risks associated with extended mate-searching can lead to the assumption that females observed moving sequentially between males must be receptive and ready to mate [1]. But, there may be other reasons for a female to move between males that are not immediately clear. Females that are not searching for mates may move through a population of males if the males hold vital resources. In these cases, selection should favour female behaviours that allow them access to these resources.

To obtain male-held resources, non-reproductive females may behave as they do when seeing mates and thereby deceive males into allowing them access to male-defended resources. In the dance fly, non-receptive females inflate their abdominal pouches in order to mimic egg-carrying females and thereby cause males to give them nuptial gifts [2]. Females' sensory biases [3] could shape behavioural responses to males, whether they are seeking a mate or the resources they hold. The responses of sexually receptive and non-receptive females to male courtship may not differ because the same sensory biases mediate female behaviour in both contexts. This is especially true where territories/refuges are held by males and of limited supply.

Fiddler crabs provide a unique opportunity to study female behaviour in situations where males are both potential mates and holders of vital territories. Fiddler crab territories contain a central burrow that is used as a water source, a mating and incubation site, a heat sink and a refuge from predators. Some females wander among the males, visiting males by approaching them and putting their feet into his burrow entrance. Females visit and reject several males before choosing a mate, searching for up to an hour [4]. On some visits, the female briefly enters the burrow and stays if she elects to mate with the male, or leaves if she does not choose to mate. Territory-holding females who leave their burrows or are displaced from them by other crabs also need to acquire new burrows, most of which are held by males. Such females wander, approach and visit courting males as they seek a new burrow (list of species in [4]). In some *Uca* species, territory-holding females will mate on the surface and incubate in their own burrows (e.g. *Uca lactea* [5,6] and *U. perplexa* [7]). Other females will leave their burrows to search for a mate. These females have mature ovaries [5,6] are sexually receptive and search for and choose a mate. Taken together, these points indicate that wandering and territory-holding are not necessarily direct equivalents of receptivity and non-receptivity in females of these species. In the banana fiddler crab, *U. mjoebergi*, females will surface mate with neighbours in exchange for help in territory defence but leave their territories to burrow mate, where the last male sires most of the offspring [8,9], perhaps indicating a stronger association between wandering and receptivity in this species.

During our behavioural observations of *U. mjoebergi*, we noticed that females that had been evicted from their territories (by other crabs or displaced experimentally) moved through the population of courting males in the same manner as mate-searching females, visiting and rejecting multiple males before settling into a new burrow. In this study, we examine the behaviours and mate preferences of presumably non-receptive females occupying and defending their own territory and wandering females found moving through the population of courting males, visiting and leaving multiple males sequentially.

Like other fiddler crabs, *U. mjoebergi* live in large, high-density mixed-sex populations on inter-tidal mudflats, defending burrow-containing territories. The crabs use the territory around the burrow (±10 cm diameter) for feeding and courting. The mating period occurs over 5 days in each 14-day tidal cycle during which males remain on the surface, courting females by waving their large claws. Mating occurs underground within an hour of the pair entering the burrow. After mating, the male stays underground mate guarding for 1–4 days until egg extrusion (after which a female cannot re-mate). Then, the male reseals the female in the burrow for another 16–20 days, until nocturnal spring tide when she releases her pelagic larvae into the water.

During the mating period, wanderers and residents are the two types of females that can be found in a population of fiddler crabs. Wanderers are females that have left their territories and move through the population of courting males, visiting up to 13 males [10]. Given their behaviour, we hypothesized that most of them should be sexually receptive, as they appear to be looking for mates. We acknowledge some of them could have been territory-holders evicted from their burrows prior to capture. We do not know the rate of female eviction or territory abandonment in this species. Residents are the females that occupy and defend a territory of their own. In our study, we caught wanderers and residents and displaced them on the mudflat and observed their behaviour as they moved among the males (male visits, empty burrow visits, etc.) We observed the behaviour of the males the wanderers and residents visited to determine whether males could discriminate between these female types (as they do in a pillar-building sister species, *U. beebei* [11]). In wanderers and residents that mated, we examined whether or not they produced a clutch of eggs (whether they were sexually receptive or non-receptive). We also tested the preferences of wanderers and residents for male traits by using robotic crabs mimicking the natural waving pattern of males. We predicted that:

(1) Significantly more wandering females should display clear preferences for particular male traits than resident females.
(2) Wandering females should be significantly more likely to select a mate and remain underground with him in his burrow, while resident females that we displace should take up residence in an empty burrow rather than with a male.
(3) Wanderer females should be more likely to produce a clutch of eggs than resident females.
(4) Wanderer females should be more likely to be allowed access to males' territories while resident females should be chased away by the resident males (as in *U. beebei* [11]).

## 2. Material and methods

We studied a population of banana fiddler crabs at East Point Reserve, Darwin, Australia (12°24′32′′S; 130°49′50′′E) in October–November of 2007, 2011 and 2012. We compared the behaviour of wanderers and residents in this study.

### 2.1. Robotic crab trials

We caught 20 wandering (observed walking across the mudflat) and 20 resident females (observed digging and eating in and around a single burrow) and tested their mating preferences using robotic crab models [12–14]. The custom-built robots are run from a central control box that decodes sound files transmitted from a portable CD player. Each robot is encoded by a unique frequency and relay switches in the control box supply current to the appropriate robot to activate a wave. The robotic crab units have an internal two-cam system (GWServo S03N 2bb) that controls the movement of a metal arm that exactly mimics the male courtship wave. The motor is enclosed in a plastic container that we bury in the sediment so that only the metal arm protrudes. A replica claw is attached to the arm. It is constructed from Hydrostone in a latex mould of a real claw, and painted with Dulux model paint that falls within the natural colour range [15].

On capture, the female was measured and held in a container with seawater until she was placed in the arena under a small, inverted plastic cup. Two robots were positioned 20 cm away from the female, both directly facing her and 5 cm apart. The female was remotely released 2 min after the robots had been activated. A positive response was scored if the female moved directly and steadily towards one robot and either touched it or came within 5 cm of it. We eliminated trials in which the female dashed immediately after release, moved to the edge of the arena or sat still for more than 5 min. Each female was tested once in each of three experiments. Because females naturally approach and leave several males before selecting a mate, it is not unreasonable to test them in three experiments [16,17]. After testing, we released the females back onto the mud flat to continue their search for a mate (if they were wandering females); or back into their original burrows (if they were resident females).

In the first experiment, we presented both the wandering and resident females with a choice between a large and a small robotic crab: the claw lengths were 24 mm and 18 mm. Both waved synchronously at the rate of 8.4 waves per min. In the second experiment, the female was given a choice between two wave rates: both claws were 18 mm long (the population average) [18] but one waved at 4.2 waves per minute and the other waved at 16.8 waves per minute (they waved synchronously every fourth wave and then the ‘slow’ robot missed three waves). Synchrony prevents any effect of wave leadership as this is known to affect female mate choice [17]. In the third experiment, females were given the choice between a leader and a follower: both claws were 19 mm long, both waved at the same rate (8.4 waves per min), but the leading wave started 1.8 seconds before the following wave (half the duration of the wave). The order of trials, positions of robots, and positions of stimuli were alternated between females.

### 2.2. Natural observations: females

We collected 59 females (32 wanderers; 27 residents), measured them, and released them individually onto the mudflat at least 2 m away from their capture point, and observed their behaviour until they had secured a new burrow. Immediately after release, the female would often dart into the nearest burrow. To avoid documenting this escape response, we did not begin recording the female's behaviour until the female emerged from this burrow and began moving across the mudflat. We followed the females from at least 1.5 m away to avoid disrupting their normal behaviours [10]. From the time of release to the securing of a new burrow, we noted the number of times that a female visited (approached, put her legs into, or fully entered the burrow) a waving male, a non-waving male or an unguarded burrow. We defined an unguarded burrow as one with no surface-active crab; these burrows may be empty (which is rare) or they may be occupied by a resident that is underground. To an approaching female, the burrow would appear unguarded until she reaches (and briefly enters) the burrow shaft. We reserve the term ‘empty burrow’ for those that were unoccupied when the female visited them. We also noted the time it took for the female to secure her new burrow.

A female could secure a new burrow in one of two ways: (i) she could visit a courting male and remain underground with him after he sealed the burrow entrance with a sand plug (this indicates a mating); (ii) she could find and occupy an empty burrow. Females very rarely dig new burrows and, unlike males, they do not fight residents and evict them in order to steal their burrows.

### 2.3. Natural observations: males

We captured 20 wandering and 20 resident females and released them individually onto the mudflat at least 2 m away from their capture point. We documented the second visit of the female to a waving male (we did not document the first approach to avoid the initial female escape response). We noted the total number of waves that the visited male gave to the female and the total number of males that waved at the female during her approach to the visited male. We also noted whether males gave aggressive waves (these are choppy vertical waves that are easily distinguished from the courtship wave) and whether the male allowed the female to enter his burrow.

### 2.4. Clutch production

In *U. mjoebergi*, females surface mate with neighbours [8] and wander to select their ‘true’ mate. Because mating occurs primarily underground in this species, a female's mating receptivity can only be determined by whether she produces a clutch. So, if a female stayed with a male, we marked the burrow by dropping a small coloured bead into the burrow shaft and then put a numbered plastic collar (10 cm diameter plastic ring, 5 cm high) around the burrow entrance to prevent either crab from escaping. We checked the burrows each day until the male finished guarding the female, left the burrow and was found on the surface within the collar. We dug the burrow up and collected the female to check if she had produced a clutch of eggs. We only used females that we found in a burrow that had a bead of the appropriate colour (to be certain that we had excavated the correct burrow). Burrows are wide at the bottom, so beads dropped down the narrow shaft do not block crabs from entering or exiting. After the crabs were excavated, we immediately placed the female in an artificially created burrow to continue incubation. If, by the end of the neap tide period, the male had not left, we dug up the burrow as above.

### 2.5. Female scare response

We documented the number of times that a female returned to a male's burrow after a simulated predator approach. We captured resident females (*n* = 20) and released them onto the mudflat more than 1 m away from their capture site. We followed each female until she had visited, rejected and moved away from a courting male. When the female had moved far enough away that she was closer to another male's burrow than to the burrow of the courting male she had just rejected, we flew a model of a bird over her. The plastic model of a small avian predator (body length = 13.5 cm, wingspan = 19 cm) was attached to a long bamboo pole (3 m) and was swooped over the focal female at a height of ±50 cm above the sediment. The female's subsequent behavioural response was scored as (i) returned to the rejected male's burrow; (ii) entered the nearest burrow to her or (iii) froze.

## 3. Results

### 3.1. Robotic crab trials

When given a choice between a large and a small-clawed male, both resident and wandering females strongly preferred the larger claw. Residents selected the larger claw in 19 of the 20 trials (binomial test *p* < 0.001); wanderers selected the large claw in 18 of the 20 trials (binomial test *p* < 0.001). There was no difference in these two proportions (Fisher's exact test: *p* = 1.00).

When given a choice between two robotic males that waved at a fast or a slow rate, both types of females strongly preferred the faster waving male. Wandering females selected 17 fast/3 slow robots (binomial test *p* < 0.001); resident females selected 19 fast/1 slow robots (binomial test *p* < 0.001). There was no difference in these two proportions (Fisher's exact test: *p* = 0.61).

When given a choice between a leading and a following robotic crab, both types of females strongly preferred the leading wave (wandering females selected 40 leading wavers and 10 following wavers; binomial test *p* < 0.001); resident females selected 38 leading wavers and 12 followers (binomial test *p* < 0.001). There was no difference in these two proportions (Fisher's exact test: *p* = 0.81).

### 3.2. Natural observations: females

Wandering and resident females did not differ in the way they secured new burrows. No female dug a new burrow; most females occupied empty burrows (wanderers: 56%, 18/32; residents: 74%, 20/27); and fewer females stayed with a male after entering his burrow (wanderers: 44%, 14/32; residents: 26%, 7/27). The proportions of wanderers and residents that mated were not statistically significantly different from each other (14/32; 7/27; Fisher's exact test *p* = 0.18).

It took females about half an hour to secure a new burrow (wanderers: $\bar{x}\;\pm s.d.=31.85\pm 23.85\;\min$, *n* = 32; residents: $\bar{x}\;\pm s.d.=30.78\pm 18.76\;\min,$ *n* = 27). They visited about seven unguarded burrows (wanderers: $\bar{x}\;\pm s.d.=5.21\pm 6.44,$ *n* = 32; residents: $\bar{x}\;\pm s.d.=8.74\pm 7.45,$ *n* = 27); four waving males (wanderers: $\;\bar{x}\;\pm s.d.=5.03\pm 7.14,$ *n* = 32; residents: $\bar{x}\;\pm s.d.=4.34\pm 6.12$, *n* = 27); and very few non-waving males (wanderers: $\bar{x}\;\pm s.d.=0.50\pm 0.92,$ *n* = 32; residents: $\bar{x}\;\pm s.d.=0.30\pm 0.72,$ *n* = 27).

Data for trial durations and female size were normally distributed (Kolmogorov–Smirnov test against a normal distribution: duration: *Z* = 0.84, *p* = 0.48, *n* = 59; female size: *Z* = 0.61, *p* = 0.85, *n* = 59). Data on the number of visits to waving males, non-waving males and unguarded burrows were not normally distributed. We could normalize two of them using a log_10_ transformation (log_10_ visits-to-waving males: *Z* = 0.102, *p* = 0.25, *n* = 59; log_10_ visits-to-empty burrows: *Z* = 0.80, *p* = 0.55, *n* = 59). Females visited very few non-waving males ($\;\bar{x}\;=0.41\pm 0.83,$ *n* = 59) and there were too many zero values in the dataset to adequately transform the data to a normal distribution. We therefore excluded this variable from our analyses.

We coded females in two ways: (i) whether the female was a wanderer or a resident; and (ii) whether the female stayed underground with a male (mated) or occupied an empty burrow (unmated) at the end of the trial. We did this because there is a possibility that the females we had found wandering on the mudflats were either searching for a mate or were females that had been evicted from their own burrows and were searching for a new burrow. Similarly, for resident females we randomly caught residents so some of them may have had ripe ovaries and would have gone mate-searching that breeding cycle, and some may have had unripe ovaries and would be burrow-searching. We therefore have four female categories: wanderers that mated (*n* = 14); wanderers that did not mate (*n* = 18); residents that mated (*n* = 7); residents that did not mate (*n* = 20) (table 1).

**Table 1.** Descriptive statistics for the four female types: the time it took to secure a new burrow (duration, min); the number of waving males visited (male visits); the number of visits to unguarded burrows (empty burrows); the size of the female (female size; carapace width, mm). The values are presented as $\bar{x}\;\pm s.d.$ (*n*) and are the untransformed total number of events per female.

female type	duration	male visits	unguarded burrows	female size
wanderer mated	36.14 ± 25.10 (14)	5.14 ± 4.05 (14)	5.86 ± 7.94 (14)	8.78 ± 1.01 (14)
wanderer unmated	28.51 ± 22.99 (18)	4.94 ± 8.97 (18)	4.72 ± 5.19 (18)	8.36 ± 1.41 (18)
resident mated	33.32 ± 21.85 (7)	6.29 ± 7.48 (7)	4.57 ± 6.65 (7)	8.34 ± 0.55 (7)
resident unmated	29.89 ± 18.09 (20)	2.55 ± 2.80 (20)	10.20 ± 7.30 (20)	8.37 ± 1.01 (20)

We ran a multivariate general linear model (MANOVA) with duration; log_10_(number of visits to waving male + 1); log_10_(number of visits to unguarded burrows + 1) as dependent variables; mated/unmated and wanderer/resident as fixed factors; and female carapace as a covariate. The female size did not differ between the four categories (Wilks' Lambda *F* = 0.95, *p* = 0.43). The duration of the trial also did not differ between any female categories and did not differ with female size (all *p* > 0.13). The interaction between mated/unmated and resident/wanderer was not significant (Wilks' Lambda *F* = 0.94, *p* = 0.34). *Comparing wandering and resident females*: there was no difference between wandering and resident females in the number of unguarded burrows or waving males that they visited (Wilks' Lambda *F* = 0.91, *p* = 0.18). *Comparing mated and non-mating females*: females that mated differed from females that did not mate (Wilks' Lambda *F* = 0.73, *p* = 0.001). Mated females visited more waving males than non-mating females. Mated females (*n *= 21) visited 5.52 ± 5.27 waving males while unmated females (*n *= 38) visited 3.68 ± 6.51 (*F* = 4.03, d.f. = 1, *p* = 0.05). Mated females also visited fewer unguarded burrows than non-mated females. Mated females visited 5.43 ± 7.39 unguarded burrows while unmated females visited 7.61 ± 6.88 unguarded burrows (*F* = 4.60, d.f. = 1, *p* = 0.04) (table 1).

In summary: wandering and resident females did not differ in the number of waving males they visited, the number of unguarded burrows they visited, the duration of their search or the size of the female. Females that ended up mating did differ from those that occupied empty burrows: they visited more waving males and fewer unguarded burrows. However, it is important to note that unmated females visited and left the burrows of multiple males ($\bar{x}=3.68\pm 6.51,$ *n* = 38).

### 3.3. Natural observations: males

Males gave a mean of 3.85 waves to the approaching female and there was no difference between the number of waves given to wandering and resident females (wandering females: $\bar{x}=3.95\pm 2.93,$ *n* = 20); resident females: $\bar{x}=3.75\pm 2.67$, *n* = 20; Mann–Whitney *U* = 198.0, *p* = 0.97). An average of 3.12 males waved at the approaching female, and this did not differ between wandering and resident females (wandering females: $\bar{x}=2.85\pm 1.42,$ *n* = 20; resident females: $\bar{x}=3.40\pm 2.62,$ *n* = 20; Mann–Whitney *U* = 189.5, *p* = 0.78). No males gave aggressive waves towards the female and all males allowed the female entry into his burrow.

### 3.4. Clutch production

Resident and wandering females that remained in a male's burrow and were successfully recovered were documented as having produced a clutch of eggs or not. Because it is difficult to recapture the pair of crabs after mating (success rate of approx. 35%), we supplemented the data by following additional females (both wanderers and residents) using the same method above. Of the 24 resident females we recaptured, 10 had produced a clutch of eggs (42%). Of the 23 wandering females, 18 had produced a clutch (78%). A likelihood ratio test showed these to be significantly different (*Χ*^2^= 6.74, d.f. = 1, *p* = 0.02). Wandering females were more likely to produce a clutch of eggs than were resident females. However, it is important to note that 42% of resident females produced a clutch of eggs, indicating that they successfully mated with the male they selected after we caught them from their own territories and released them onto the mud flat.

### 3.5. Female scare response

Of the 20 females that had left a visited male's burrow, 17 of them returned to that burrow when they were approached by a model predator, even though they were closer to another male's burrow. Two of the females approached the closest burrow and one female froze until the predator had passed her (binomial test 17 : 3: *p* = 0.002). Most females returned to the last burrow they had visited when they were evading potential predators.

## 4. Discussion

When a female moves between and interacts with courting males, we tend to interpret this behaviour as an indication that she is sexually receptive and looking for a mate. Our study indicates that this is not necessarily the case. We examined two categories of females, wanderers that were moving through the population of courting males and residents that were occupying and defending their own territories. We expected that when we displaced the wanderers and the residents, the wanderers would exhibit more mate-searching behaviours than resident females. However, we found that wandering and resident females were nearly identical in mate-searching behaviour. They preferred the same male traits (large claws, fast waves, leading wavers), visited the same numbers of males and unguarded burrows, avoided non-courting (non-waving) males, and stayed in males' burrows in equal proportions. Males also behaved the same way towards wanderers and residents, suggesting that they were unable to differentiate between the two categories of females. Why, if not actively looking for a mate, would resident females show the same preferences and behave in the same way as wanderers, especially given the high risks associated with mate-searching?

We suggested that females could be deceiving males in order to gain a resource. Consistent with our data, the female's sensory biases could have shaped their behaviour, causing them to move through the population in the same way, whether they are searching for a mate or for some other resource. The most important resource a fiddler crab can have is a burrow: it is the only source of water at low tide, it acts as a heat sink, and shelter from predators and the high tide. If a crab does not own a burrow when the tide comes in, it will almost certainly die. The benefits of having access to a burrow may favour females that exhibit the same responses to male courtship whether or not they are seeking mates. In this way non-receptive females may deceive males into allowing them to approach and enter their burrows as males do for receptive females. In our study, the males allowed burrow access to all females equally (wanderers: 44%, 14/32; residents: 26%, 7/27). Although our sample sizes were modest, we should have been able to detect a large effect (power analysis for an effect size of 0.9 at *α* = 0.05 and *n* = 32; the power would be 0.95). However, our other data also support this lack of discrimination (males behaved the same towards wanderers and residents). Clearly, males paid a cost for this. When they allowed access to resident females, only 42% of them produced a clutch of eggs. However, wandering females were more likely to produce a clutch (78%).

Although it is costly for males to be deceived, it may be best for them to court all females rather than making a potentially costlier error of turning away a true mate-searching female [19,20]. A similar case exists in the spider *Paratrechalea ornata* where males offer nuptial gifts to attract females; however, 70% of the males in the population offer silk-wrapped ‘worthless gifts’ not containing food items but bits of inedible insect parts or plant matter. Females accept these ‘gifts’ by giving males mating access, and do not discover the true nature of the gift until the mating attempt has begun [21]. The female's benefit in selecting a mate with a gift outweighs the cost of selecting one without a gift so that, on average, selecting a mate with a ‘worthless’ gift is better than selecting one with no gift at all. In both the crabs and the spiders, the receiver response to the signal is probably adaptive and is not selected against, allowing the deceivers to continue to benefit at a cost to the receivers [22].

Our data suggest that selection has shaped the behaviour of mate-searching and burrow-searching females in the same way. Both types of females display the same responses to courting males irrespective of what they are searching for as the presence of a male reliably signals the location of a burrow. Either females evolved their responses in the context of finding a new territory and were later co-opted into the mate-searching context or vice versa [23]. Data from other *Uca* species, especially those in which the courting males build landmarks such as hoods (*U. terpsichores*) or pillars (*U. beebei*) beside their burrows suggest that the females use landmark orientation to navigate across the mudflat [4,24,25]. These landmarks provide a place to hide from predators [4,24,26–28]. Consistent with this work, we show that females return to the last-visited burrow when they are threatened by a potential predator, suggesting that the females move from burrow to burrow in a way that allows them to always have a path map to a nearby refuge. We know that fiddler crabs use leg odometry to integrate their movements [29]. Mate-searching females make frequent adjustments to their body orientation so that they keep their transverse axis aligned with the bearing of the last-visited burrow [25]. This enables them to quickly return to that burrow in a straight line. As the females move further away from the last-visited burrow, there is a point at which they ‘break’ the connection to that burrow: this is visible as the female abruptly changes her body orientation and her direction of movement to approach the next male to be visited, following him back to his burrow [4,25]. As with other fiddler crabs, the *U. mjoebergi* females' territory-seeking behaviours have been co-opted to shape their mate-searching behaviour (or vice versa). When females are searching for either a new territory or a mate, the most efficient way to do this is probably to move from male to male, always keeping a path map to the last-visited burrow in case of a predatory threat.

What evidence do we have that non-receptive females are exhibiting deceptive behaviours consistent with their sensory biases [3]? If non-mate-searching females are mimicking mate searchers in order to gain access to their burrows, then they should not exhibit a preference for ‘attractive’ male traits because male quality is irrelevant for females only interested in a burrow to shelter in. Here, we show that all females preferentially approached large, fast waving males that produced leading signals. While non-receptive females are still deceiving the males, this consistent preference for male traits adds weight to the idea that females' sensory biases are used in any contexts involving moving around on the mudflat.

The only behavioural differences we could detect were not between wanderers and residents, but between females that ended up in a burrow with a male at the end of the trial (mated) and females that did not. Females that eventually mated visited more males and fewer unguarded burrows, consistent with results reported for two related species, *U. pugilator* [30] and *U. beebei* [11]. A study of a third species, *U. terpsichores* (formerly *U. musica*), showed that receptive females (females that mated) visited more males than non-receptive females [25]. A potential explanation for the lack of difference in behaviour between wanderers and residents is that some of the females found wandering on the mudflat (and hence classified as mate searchers) were not actively searching for mates. They may have been ejected from their burrow or chosen to leave their burrow due to neighbour interactions (see [5]). These females would not have necessarily been ‘true’ mate searchers as they potentially would have already mated, and therefore would effectively be territory searchers. Second, some of our resident females may have been nearly ready to leave their own burrows in search of a mate when we displaced them to a group of courting males. These former residents, upon displacement, would ‘turn into’ mate searchers [5], and would have finally ended up choosing a male to mate with. Whatever the explanation, what is clear is that while moving across the mudflat, whether territory-searching or mate-searching, female *U. mjoebergi* behave in way that is indistinguishable to researchers or to conspecific males.

This is interesting for many reasons. These results suggest that in this species, male visits and rejections cannot be used as an indicator of female mate choice. In a classic, well-cited mate sampling paper, Dale *et al*. [31] claimed that the distinction between active female choice and passive attraction could only be solved by direct observation of a female visiting and rejecting a potential mate. Here, we show that that is not necessarily true: the rejection of a potential mate is not sufficient evidence of active mate choice. We observed classic visit and reject behaviour by resident females that were not looking for a mate. Consistent with other studies of *Uca* species [4], we suggest that all females behave in the same way because it is the safest way to move through the population and the selective pressures for territory-searching and mate-searching are nearly identical in these species, as males are by and large conspicuous signals of the presence of burrows. In a pied flycatcher study, the authors [31] caught females and released them in another population to observe their subsequent mate sampling behaviour. Sixty per cent of the females did not stay and mate although most had visited multiple males before disappearing. It was assumed that these females left the area and selected a mate elsewhere. This may be true; however, this species is well known for settling close to their release site [31–33]. It is possible that the nine pied flycatcher females that left the area were not ready to mate and that their ‘sampling’ behaviour was for other reasons that we have yet to determine.

In conclusion, we have shown that (i) visiting and rejecting several males is not the defining feature of female mate choice; (ii) the way that all females move through the population of courting males (by approaching and leaving a succession of burrows) is an adaptive anti-predator behaviour that is useful in the contexts of mate-searching and territory-searching. Additionally, we suggest that researchers look more closely at the behaviour of females that are moving through a population of courting males as mate-searching is not the only possible interpretation of their behaviour. Rather than looking at apparent mate-searching behaviour as an outcome of social selection (*sensu* [34]) we should also acknowledge there may be ecological selection at work.

## Supplementary Material

Data for this manuscript.

## Data accessibility

The data relevant to this manuscript are available as the electronic supplementary material.
